# The Hospital. Nursing Section

**Published:** 1905-10-28

**Authors:** 


### The Hospital.

### Nursing Section.

Contributions for this Section of "The Hospital" should be addressed to the Editor, "The Hospital" Nursing Section, 28 & 29 Southampton Street, Strand, London, W.C.

No. 996.—Vol. XXXIX. SATURDAY, OCTOBER 28, 1905.

### Notes on News from the Nursing World.

### OUR CHRISTMAS DISTRIBUTION.

The advent of cold weather at an unusually early pe iod is likely to enhance the distress which usually prevails in the winter, and we are not surprised to receive from the secretary of a well known Maternity Charity in the East-end of London, an earnest appeal to us to remember that institution when we are sending out parcels at Christmas. We feel sure that our kind readers will enable us to comply with this and similar requests which always reach us at this time of the year; and we hope that every week in November we shall have to announce the arrival of a goodly number of contributions. We have received parcels from L. R., Buxton; and Miss Margaret Woodhouse, Teddington. All contributions should be addressed to the Editor, 28 and 29 Southampton Street, Strand, London, W.C., with "Clothing Distribution" written on the outside.

### OUTSIDE INTERESTS FOR HOSPITAL NURSES.

The probationers at the Chelsea Hospital for Women are encouraged in every way possible to have outside interests as well as to devote their best attention to their work in the wards. Last winter a singing class was got up, and Miss Florence Christie, the well-known singer, kindly taught class-singing for many weeks every Wednesday evening, from eight-thirty to nine-thirty. For the present winter the nurses are fortunate in having the advantages of lessons in physical training under Miss Roffel, from the Polytechnic College, in Manresa Street. This Wednesday evening hour is much looked forward to from week to week. The little class of suitably-attired gymnasts is most interesting to the matron, who has always as strongly advocated wholesome and improving recreation as she has required sound and earnest work during the hours of duty. Some nurses may think gymnastic exercises a strange form of pleasure at the end of a day's hard work, but there are muscles little used in ordinary life which become highly developed with proper exercises. The making of the dresses suitable for physical exercises is also a source of pleasure, calls forth ingenuity, and encourages a useful employment of time when the nurses are off duty in the evening.

### THE NEW MATRON OF THE NATIONAL HOSPITAL.

The vacant post of Lady Superintendent of the National Hospital for the Paralysed and Epileptic was filled up on Tuesday by the appointment of Miss Isabel Lawrence. Selected from a large number of applicants, Miss Lawrence has had an uninterrupted nursing career since February 1889. Trained at Crumpsall Infirmary, Manchester, she subsequently had charge of the male and female wards at the Cancer Hospital, Brompton. Her next post was that of charge nurse of the Bentinck Ward of the Institution to which she now returns as head of the Training School. She held this post for a year, and after a year of private nursing served as sister of the Chandler Ward at the National Hospital. In 1902 Miss Lawrence was chosen for the responsible post of Lady Superintendent of the Meath Home of Comfort for Epileptics at Godalming. Her experience, therefore, should qualify her in the fullest possible manner for her new appointment.

### EXAMPLE TO AMERICAN NURSES.

It will be learnt with interest by many English nurses that Miss Isabel McIsaac has offered herself for service in the Army Nurse Corps of the United States. That Miss McIsaac, after twenty years of hard work, should be ready to emerge from the retirement which she has so well earned in response to the call of her country, ought to have a stimulating effect upon patriotic American nurses, and shame some of the younger women into placing their names on the Volunteer Eligible List. How slowly the enrolment is going on may be judged by the fact that in a month the number of recruits has only risen from forty-one to forty-three. It may be true that if the need arose thousands of American nurses would be clamouring for an opportunity to serve, but their assumed readiness to fulfil an obligation in the future does not absolve them from a duty in the present.

### A REMINISCENCE OF HENRY IRVING.

Amongst the many who were present at the house of the Baroness Burdett-Coutts in Stratton Street last week to bid farewell to the mortal remains of Sir Henry Irving was a sister from one of the large London hospitals. She had simply presented her card at the door and been admitted. As she stood overcome by the solemn hush and the perfume of the thousands of flowers massed around her thoughts went back many years, to a time long ago when an Irish girl had come over from the Emerald Isle to consult an eminent London oculist. One eye was at that time nearly blind; but this did not lessen the intense desire of the girl, if possible, to get to the Lyceum, and after some difficulty a seat was procured close enough to allow even a person with defective sight to witness the beauties of "Faust." The evening came, but when the girl arrived at the theatre she found that her seat, owing to an error, had been assigned to someone else. In her distress she told her story to a gentleman standing by, who, bidding her wait, moved rapidly away. In a few moments he returned, asking her to follow him, and after guiding her along many back passages, stopped at a little door. "This," he said, "is Mr. Irving's own box. I told him of your trouble, and he wishes you to sit right in front, that your eyes may not be unduly strained." Thus the great actor's kindly thought doubled the pleasure of that performance for the future hospital sister.

### DEVONSHIRE NURSES FOR DEVONSHIRE POOR.

At the opening of the new Poor-law Infirmary attached to the Exeter Workhouse, in which there will be accommodation for 150 patients, Sir Thomas Dyke Acland congratulated his hearers on the fact that the nurses were carefully provided for. He considered this an important point and said that he was glad to see that in the infirmary there were at least likely to be five or six nurses "to keep each other company." He went on to express a hope that the institution would prove a training school for Devonshire girls to nurse Devonshire sick poor, and maintained that as the object of the Victoria Jubilee Institution was the same as that of those who built the infirmary—namely, the efficient care of the sick poor—it should be possible to amalgamate the two.

### THE WORKHOUSE NURSING ASSOCIATION AND THE COMMISSION.

We are glad that the Workhouse Nursing Association have shown their vitality by sending a resolution to the Prime Minister and the President of the Local Government Board, suggesting that in view of the increasing number of sick cases in poor-law institutions, at least one woman possessing a wide practical acquaintance with the working of the Poor-law in relation to sick and infirm inmates in both large and small workhouses, should be appointed on the Royal Commission. But we imagine that this is a case in which pressure is not needed.

### NURSES AND CHEMISTS.

Our attention has been called by a Leeds correspondent to the practice which, he maintains, goes on among nurses of obtaining articles at wholesale or medical prices for their patients, when, in the ordinary course, the latter would have to pay full prices. He affirms that it is most difficult for the retail pharmacist in these days of competition to live, and he thinks it is not right that nurses should add to the difficulties. We cannot defend the action which he condemns. It is one thing for a nurse who makes a purchase for herself from a chemist to ask for it at a reduced price, and quite another for her to try to use her professional position in order to save the pocket of a patient. This, we think, is not legitimate, and nurses with a keen sense of fairness should set their faces against it.

### AN INCIDENT AT CHICHESTER.

At a recent meeting of the Chichester Guardians a letter was read from a probationer—who had gone to see her father who was ill—asking for a long vacation or to be released by the Board from her engagement, which does not expire until February. In another letter to the superintendent nurse, which was also read, the probationer apparently anticipating a refusal, said "Let the Guardians do their worst." This appears, on the face of it, to belong to the class of communication which should neither be written nor read, but it is due to the probationer to give her version of the matter. Some weeks before she left the Chichester Workhouse she asked to be allowed to resign her post, but received no answer. At length she heard that her father had suffered a serious relapse and was believed to be dying. At this point the superintendent nurse undertook to see the Chairman of the Board of Guardians, in order to obtain permission for her to go to him without delay. For five days the probationer waited for permission, and was then told that "she could not go unless her father was dying." Two days more passed, and she received a telegram from home—"Father worse. Come to-day," and she went at once without again asking leave. Subsequently she wrote to the Guardians expressing regret for the inconvenience caused, and offering to return as soon as possible. It is added that her father was slowly dying. While we think it is a matter of regret that a probationer should even appear to be prepared to set the Guardians at defiance, we do not see how a summons to the bedside of a dying parent could be disregarded, and it is a pity that the Guardians delayed complying with such a natural request.

### NORTHAMPTON GENERAL HOSPITAL.

By permission of the authorities, Mr. Louis H. M. Dick, Secretary of the Royal National Pension Fund for Nurses, delivered an address at the General Hospital, Northampton, on Monday evening last week on the aims and objects of the Fund. The Matron, Miss Densham, was present, and amongst an audience numbering more than forty were Miss Lunn, Superintendent of the Victoria Nurses' Home, Miss Stride, of the Nursing Home, Hazelwood Road, and Miss Taylor of the Convalescent Home, Weston Favel. Amongst other points Mr. Dick mentioned that as the Fund became better known it increased rapidly in popularity, which was evidenced by the fact that already this year over 1,000 policies had been issued. In concluding his address he thanked the governing body of the hospital for so kindly having lent the Boardroom for the purpose of the meeting, and Miss Densham for the trouble which she had taken in the matter.

### THE BISHOP OF MANCHESTER ON THE PARISH NURSE.

Preaching on behalf of the Parish Nurse Fund in connection with St. Clement's, Greenheys, the Bishop of Manchester said that as a parish clergyman he had known many lives saved, others prolonged, and much needless suffering avoided by just that skilled help which a trained nurse could give. With that knowledge he had no hesitation in commending the fund; but he felt that if some whom these nurses had visited in their sickness could stand where he stood, one story from their lips would stimulate help in the cause more than any words of his.

### THE NURSING OF FEEBLE PAUPERS.

The authorities of the Workhouse at Cardiff—which has just been worthily elevated to the dignity of a city—think that there is no urgent need to increase the staff of one male nurse who looks after a hundred patients in the workhouse by day, may do well to observe that the death of an old woman at Withington Workhouse, Manchester, who succumbed to heart failure accelerated by the fact that she had eight fractured ribs, prompted the jury at the inquest to express their opinion that more attention should be given to the old and feeble inmates of the wards. It is the old story. The individual members of the nursing staff who had the deceased under their care do not appear to have been to blame; but there do not seem to have been enough of them. Three weeks before her death the old woman of eighty-four had fallen out of bed, though the medical man was of opinion that the injury had not taken place then. At all events, the incident emphasises the importance of sufficient nurses being employed to look after such helpless patients at Withington and elsewhere.

### PROMOTIONS FROM WITHIN.

Through an unfortunate error in a "Note" last week, referring to promotions at Charing Cross Hospital, a portion was omitted which destroyed the sense of the conclusion. In the omitted part we drew attention to the fact that we are always glad to learn of these promotions from within, as their interest to the friends of nurses is as great as the appointments which include a change of locality.

### QUEEN ALEXANDRA'S MILITARY NURSING SERVICE.

Miss M. Plaskitt, staff nurse in Queen Alexandra's Imperial Military Nursing Service, has been posted to the Royal Herbert Hospital, Woolwich. Miss M. Kendall, sister, has been transferred to South Africa from Woolwich, and Miss K. Pearse, sister, to South Africa from Chatham. Miss C. M. Williams has been appointed staff nurse provisionally.

### AN INVITATION TO TIRED NURSES.

With great kindliness, Mrs. C. S. Henry will throw open her Recreation Home at Parkwood, Henley-on-Thames, two miles from Wargrave Station, from the beginning of November to the end of March to trained nurses. The Home was originally established for children, for whom it is admirably suited during the spring and summer months. It is, in fact, so much appreciated that from March 30 to the middle of October there was not an empty bed. As, however, it has been found desirable to close it after October to children, Mrs. Henry desires that it should be of use to nurses requiring rest or change. The Home is exceptionally pretty, being an old farmhouse standing in its own pleasant grounds. It is fitted with every modern convenience, and there are practically no rules, the object of Mrs. Henry being to make it a real rest and relaxation to the nurses who have the good fortune to partake of her hospitality. All the visitors are her guests, and particulars as to when they can be received and the length of stay may be obtained on application to Miss Shaw, the Matron of the Home.

### IRISH MATRONS' ASSOCIATION.

A meeting of the Irish Matrons' Association was held last week at 86 Lower Leeson Street, when final arrangements were made for the examination of the "Incorporated Society of Trained Masseuses" to be held in Dublin in February 1906. Classes are now being formed at many of the hospitals and by private teachers to prepare pupils for this examination. Intending candidates should apply for particulars to the Hon. Sec., Irish Matrons' Association, 86 Lower Leeson Street, Dublin.

### EXAMINATION AT WEST HAM UNION INFIRMARY.

Following the second examination of probationers at West Ham Union Infirmary, Mr. T. H. Openshaw, C.M.G., reports that the general efficiency and knowledge of the nurses of surgical and medical nursing were very excellent indeed. He is satisfied with the proficiency of all the nurses. The lowest number of marks was 134 out of a total of 225. Nurse Williams obtained the highest marks, with Nurse Filley quite close to her as second. Mr. Openshaw thinks that their extraordinarily exact knowledge of nursing, their skill in bandaging, and the general medical knowledge of the candidates reflect the highest credit upon the care with which they are taught by the matron and the medical officers. The names of the nurses examined are G. Williams, E. M. Filley, J. F. Lumsden, E. Leahey, A. C. McInerney, E. Greenhough, K. McCarthy, N. McCarthy, S. Donohoe, E. Braime, H. A. Woods, R. Bush, E. Henshaw, G. Dorkins, and E. Daynes. The Guardians, in signing the certificates, expressed their appreciation of the satisfactory result of the examination. Dr. E. Vallance, Medical Superintendent of the Infirmary, has presented a fitted wallet to Nurses Filley and Lumsden for the excellent manner in which they kept a temperature chart.

### SHORT ITEMS.

A social gathering of the members of Sussex County Nursing Association, working in the western division of the county, was held recently at Old Park, Chichester. Six nurses and four members of local committee accepted Lady Gifford's invitation. A most enjoyable day was spent, and the nurses much appreciated the opportunity afforded to them of meeting old and new friends.—The South Stoneham Guardians at their last meeting passed a vote of thanks to the nurses at the workhouse, who, when two men were suffocated by gas in the institution, worked day and night without extra help and used every effort in order to save life.—Miss Florence Nicholson, staff nurse at St. Bartholomew's Hospital Convalescent Home at Swanley has been appointed organist at the same institution.

### The Nursing Outlook.

"From magnanimity, all fear above;
From nobler recompense, above applause,
Which owes to man's short outlook all its charm."

### POINTS IN A PROBATIONER'S CAREER.

There are certain points affecting the training of a nurse upon which opinion has much crystallised of late. Whilst conflict existed in regard to general questions like the importance of a three years' residence in a hospital, preliminary schools as tests to qualifications for training, and other important matters affecting every probationer alike, minor, though important, matters have necessarily been left in abeyance. Seeing, however, that in the near future every training school for nurses, which can be held to be efficient, must fulfil certain conditions, and have direct relations with other similar schools and smaller hospitals and institutions which provide material for the training of nurses, it may be well to consider a little in detail a few matters which affect the probationer and minister to the success or otherwise of the training which she receives.

All the misunderstanding and most of the objections to the three years' course arose initially from the feeling on the part of some of the most practical instructors of nurses, that a hard and fast rule of this kind was in practice likely to prove more obstructive than useful. They knew, as all experienced teachers realise, that the technical part of the nurse's work, that is the actual technicalities which she has to master to enable her to learn effectually all that experience can give her, can be taught, and indeed must be taught, in twelve months. There is of course a certain humour about the present situation in the nursing world as seen by observers of experience and knowledge, for necessarily many of the most active heads of the chief training schools and institutions, in the days when they were trained, had to be content with twelve months' probation in a hospital. Reduced to practice a three years' course means that every probationer shall not only be taught technical nursing, but that she shall devote a sufficient period, initially, to the mastery of the various branches of nursing, which can be acquired by experience through observation. Twelve months may suffice for technicalities, but two more years is not too long a time for a probationer of average intelligence to master the business of nursing, and during this period her experience, to be of any value, needs guidance and supervision. This guidance and supervision can only be adequately secured at a well organised nurse-training school, and it will be seen, as is now generally admitted, that a three years' course is essential to the making of an efficient and experienced nurse.

The fact that a nurse's knowledge, to be of value, is dependent upon experience which needs guidance and supervision, should prevent the responsible head of a great hospital from supplying the public, through its nursing institution, with the services of a probationer, and from charging for her services the fees of a fully-trained nurse. No great hospital employing a large staff of nurses, and having a training school attached to it, can properly discharge its duties to the public and to the nurses, and at the same time have any sufficient regard to its responsibilities, if it allows its officers to supply the public with any nurses for private cases, except those who have completed their training and obtained their certificates. It is no answer to these objections for any such institution to claim, that the probationer has had experience in the particular case to which she is sent, and is so qualified to co-operate efficiently in the treatment prescribed by the practitioner in attendance. The public sends to a great hospital for a nurse, that is a fully trained and certificated nurse, and it is certainly not justifiable for any hospital to supply probationers to private families in such circumstances. Besides the practice is very unjust to the probationer herself, who, under this system, is taken away from the training school and left to her own devices whilst engaged in private nursing, where such experience as she may gain must be inadequate, to say the least, for it cannot receive guidance or proper supervision.

There is one other point which affects probationers, because if it be not clearly defined, it may hamper her future after she is qualified. It is maintained by authorities whose opinion is entitled to respect, that nurses cannot be tied up to hospitals and co-operations, that is to say that on the completion of three years' training every nurse who receives her certificate, at whatever school she may have been trained, must be left free to choose what branch of her profession she will follow. If she voluntarily joins the private nursing staff of the hospital where she was trained, or becomes a member of its staff, so much the better. But if neither course appeals to her, it must be made perfectly easy for qualified trained nurses to work independently. The higher the type of woman, and the better her education, the more certain is it that each nurse must be free to do her work on her own terms as a free agent, if she so wishes, and not necessarily as an employée of a hospital or institution, however good and generous their treatment may be. It would never do for the hospitals to retain the services of a number of dissatisfied nurses, nor would it promote the popularity and efficiency of any training school to try and enforce a regulation, whereby all probationers, on receiving their certificates, should be compelled to join the staff at the hospital's option. Perfect freedom on completion of the training is essential to both parties, and must tend to promote their mutual interests to the fullest extent.

### Abdominal Surgery.

By Harold Burrows, M.B., F.R.C.S., Assistant Surgeon to the Bolingbroke Hospital.

### II. SYMPTOMS IN ABDOMINAL DISEASE.

Before describing with detail the maladies to which each abdominal organ is liable, it is convenient to refer briefly and in a general manner to the symptoms which are most commonly met with in abdominal disease. Having thus collected the materials, it will be easier to construct in the mind's eye the images of real cases hereafter to be nursed and studied by the bedside.

### Pain.

In the abdomen, as in other parts of the body, pain is nature's chief danger signal, and in one form or another it is present at some period in almost every case of abdominal illness. Occasionally it is only a late symptom, coming, it may be, when the disease has far advanced and is past hope of cure. But very often indeed pain is the earliest sign of morbid derangement. In appendicitis, for example, the illness commences with pain, and pain is the predominant symptom throughout. But "stomach-ache" is such a common complaint, and is so often due merely to dietetic indiscretion or other trivial cause, that it may be asked, "How is a nurse to know whether it has any serious meaning or not?" The answer is simple. Neither nurse, nor doctor, nor anyone else can *know*, as a rule, unless he has looked inside the adbomen to see. It is possible for the nurse and it is her duty to form an opinion and to act accordingly, but a mistaken opinion here is a very grave matter if it leads to wrong treatment. A dose of castor oil given to a patient whose constipation is caused by a growth in his bowel may put an end to his existence. To attribute to unripe apples the pain and vomiting of a child who has appendicitis may be equally fatal. Therefore my earnest advice to nurses is this : form an opinion on every case but never act upon it, unless driven by necessity to do so, otherwise than by sending immediately for a doctor and keeping the patient quiet in bed until he arrives. Meanwhile nothing should be given by the mouth except sips of water. If the pain is very severe a hot stupe or fomentation may be applied to the abdomen; but beyond these simple measures the less that is done the better. Of course, where no doctor is within reach, as often is the case in the Colonies and abroad, the nurse may be obliged to act otherwise than in accordance with this rule; but whenever possible she should avoid all responsibilities which she is not qualified or intended to assume. In many acute lesions affecting the abdominal viscera the only hope of saving the patient's life lies in early operation; every hour counts, and procrastination is fatal.

Speaking generally, abdominal pain may be classified into four groups—namely, (1) visceral pain, (2) peritoneal pain, (3) referred pain, (4) miscellaneous.

### Visceral Pain (Colic).

The chief kind of visceral pain is that which accompanies vigorous but ineffectual contractions of a hollow viscus. This variety of pain passes under several names, according to the organ involved. In the case of the bladder and rectum it is known as vesical and rectal tenesmus respectively. Uterine pains are of the same nature. But in other situations the painful contractions are described as "colic." Thus colic may be intestinal, biliary, renal, or appendicular accordingly as the intestine, biliary system (gall-bladder and bile-ducts), kidney, or appendix is concerned. Originally the word colic was applied only to pain originating in the colon or large intestine, but the word has been found convenient, and so its application has extended to other organs.

The principal features of colic are the intermittent nature of the pain and the absence of tenderness over the abdomen.

The chief points of difference between colic (visceral pain) and the pain of peritonitis (peritoneal pain) may be observed by studying the tables of comparison given below.

| Colic. | Peritonitis. |
|---|---|
| Pain intermittent. | Pain continuous. |
| Abdomen moves with respiration. | Abdomen motionless. |
| Abdomen not tender. | Abdomen very tender. |
| Pain may be relieved by pressure. | Pain much increased by pressure. |
| Pulse probably less frequent than 100 per minute. | Pulse probably more frequent than 100 per minute. |

The treatment of colic will depend upon the cause and the organ affected, and will be dealt with later on when the affections of the various viscera are separately considered.

### Peritoneal Pain.

The general arrangement of the peritoneum and its division into a parietal and a visceral layer has been referred to in a previous article, and it was stated there that the parietal layer is extremely sensitive to injury, while the visceral layer is hardly sensitive at all. For practical purposes, therefore, it may be taken that peritoneal pain originates only in the parietal layer. If this becomes inflamed as the result of injury or of septic infection the most acute pain is caused. The outstanding characteristics of peritoneal pain are mentioned in the table of comparison between colic and peritonitis, but the subject will be constantly recurring in subsequent articles, and especially when peritonitis is being discussed.

### Referred Pain.

Just as a patient with hip disease may complain of pain in the knee, so may an individual feel pain in the abdomen when the real cause of trouble is elsewhere. Good examples of this are pneumonia and pleurisy. In some instances these processes are accompanied by severe pain in the abdomen, and it is occasionally quite easy to mistake them in the early stages for acute abdominal disease.

### Miscellaneous Pains.

Under this heading may be placed examples of abdominal pain which do not fall readily into the other three groups. Such are the pains of locomotor ataxia (tabes dorsalis) and other not very common conditions. Imaginary or pretended pains need no more than a passing reference.

### Vomiting.

As a symptom of abdominal trouble vomiting comes next to pain as regards frequency. But seeing that numerous ailments not directly affecting the abdominal organs, for example, scarlet fever and other exanthemata, injuries to the head and cerebral tumour, are often the causes of vomiting, the mere fact of emesis, considered by itself, has no special significance. The character of the ejected matter, however, may be a most valuable indication to the medical attendant as to the nature of the patient's illness, and for this reason the nurse should always preserve a specimen for his inspection. If there is repeated vomiting successive specimens should be kept.

The nurse should notice whether the vomiting is preceded by nausea and retching, or whether it comes on suddenly without any warning, and she should observe how much pain or distress is caused by the efforts.

### Other Symptoms.

Other frequent signs of abdominal disorder are an expression of anxiety, pallor, thirst, increased pulse-rate, irregularity of the bowels—diarrhœa or constipation—increased frequency of micturition or retention of urine, distension of the abdomen, and shock. The consideration of these symptoms in detail will be deferred to a future article.

*(To be continued.)*

### The Nurses' Clinic.

### THE AFTER-CARE OF OPERATION CASES IN DISTRICT NURSING. BY MISS M. LOANE.

Doctors at the present day generally arrange to perform operations as early in the morning as possible, and the plan has great direct and indirect advantages for district patients. A sleeping-draught is usually given the night before, and the patient is in such a drowsy state that all the final preparations (even an enema, if ordered) can be made without rousing him to anything like full consciousness of what is going on; thus sparing him much mental distress and anxiety. If it is impossible to keep the patient in another room until the moment of the operation, a screen should be placed round his bed.

A further advantage secured to the patient by the early hour is that the district nurse is able to remain with him for a considerable time without breaking into her night's rest.

After the operation, as soon as the patient has recovered consciousness and vomited slightly, give him half a pint of water as hot as he can drink it. This will cause him to vomit freely, and he will then have no further trouble in this respect. Without this assistance he might be frequently disturbed by slight sickness.

*Temperature.*—The temperature the first night after a successful operation will be 99° F. or 100° F., the pulse probably about 110; by the next morning the temperature will be normal, or very nearly so, and the pulse considerably reduced in rapidity. Careful watch must be kept over the temperature, and the doctor invariably warned if it reaches or exceeds 100° F.

*Diet.*—The patient must be given no food at all for four to six hours after the operation. For the first three days only cold milk should be given. Later on an adult will require three pints, and the amount is best divided into twelve five-ounce feeds, carefully heated in an enamel saucepan. A few drops of water in the bottom will prevent the milk from being burnt, but it is safer to heat it over gas, oil, or methylated spirit than over an open stove. In very poor houses a small jug can be covered with clean paper and stood in a saucepan of boiling water.

From the end of the third day until the stitches have been satisfactorily removed from the wound (probably the seventh or eighth day) give an egg beaten up in milk, light milk puddings, bread and butter cut thin and without crusts. From this time to the end of the fortnight the patient may have in addition well-made milky tea, fresh steamed fish with a little white sauce, calves' or lambs' sweetbread, tripe if very fresh and well prepared, and pigeon or chicken if obtainable. Take care that by "fish" the friends do not understand tinned salmon or dried haddock.

If all goes well, ordinary diet can be taken from the end of the fortnight, but it must be well cooked; food specially difficult of digestion must be avoided, and it must not be taken in large quantities at any one meal, but the amount of food spread more evenly over the 24 hours than is usual when people are in perfect health.

For the first three days the patient must be fed every two hours, and about three times in the night. From the end of the third day to the seventh or eighth, food must be given every three hours, and hot milk twice in the night. From the seventh day to the fourteenth food must be given every four hours, and hot milk once or twice in the night. It must be noted that in ordinary and successful operation cases very little alcohol is given. Precise instructions must be obtained from the doctor as to amount, and the circumstances in which it will be needed.

*Nursing Attendance.*—The district nurse should make careful arangements with the relatives that the nursing, exclusive of the work she does herself, shall be carried out by not more than two persons, and that except when additional help is really needed, only one of these at a time should remain in the room. Nothing can well be more disadvantageous for the patient than to be surrounded by an indefinite number of friends, advisers, and sympathisers, while the presence of even two persons in the room will probably result in an amount of conversation likely to have a disturbing and exciting effect. On the fourth day any near relative may be admitted for a few minutes, but up to the end of the first fortnight the quieter the patient can be kept, and the fewer visitors he sees, the better. The patient must not attempt to wash himself, and for at least a fortnight must be waited on "hand and foot."

It must be remembered that bedsores of a serious nature may arise within a few days, and the greatest care must be exercised from the beginning ,especially with regard to keeping the patient's skin perfectly dry, and pinning sheet, drawsheet, and mackintosh to the edges of the mattresses with large safety pins.

*Night Nursing.*—It is not always possible for the relatives to do the night nursing, but trustworthy elderly women can often be found who, with a little instruction from the district nurse, can very well undertake the work. The charge "to oblige" is a shilling a night; the usual terms two shillings.

*Bedding.*—Water beds, bed rests, mackintoshes, etc., can often be borrowed from the various religious organisations in the town. Good bedding is most desirable, but it is not safe to hire mattresses and pillows.

*Position in Bed.*—For the first week the patient must lie perfectly flat, with only one small pillow under the head. After the stitches have been removed a second pillow may be used occasionally. At the end of the second week the patient may get out of bed while it is being made, and soon after will be able to sit up for an hour, increasing the time gradually to two hours or more.

*Convalescence.*—The fact that convalescence is gradual must be impressed upon the friends. In these cases the patient may be trusted to make all the exertion of which he is capable, and they must be warned to check rather than incite his efforts. Often with the best intentions, and sometimes from shortsighted selfishness, the patient is urged to do things for which he is physically unfit, thus causing relapses, and needlessly prolonged illness.

*Change of Air.*—If possible, the patient should have some weeks' change of air, and not return to work until health is thoroughly re-established. Only too often the poor crawl downstairs directly they are allowed to leave their bed, and try to resume all their usual habits immediately.

*Fresh Air.*—How soon the patient can go out of doors depends upon the time of the year and local circumstances. A few steps might with advantage be taken in a dry, sunny garden, when to walk in a crowded, noisy street would be positively injurious. A bath-chair can often be borrowed from church or chapel organisations, or can be hired without a man for sixpence an hour. As a rule, however, these chairs are of far more use for chronic patients than for convalescents, who commonly have a rooted objection to them.

The first and last word with reference to the care of operation cases is caution.

### Training in a Hospital for Incurable Diseases.

I have often been asked the question, "Where did you train?" and when I have replied, "At the Hospital for Incurables, Edinburgh" my questioner has invariably remarked, "How very depressing!" "Not much to be taught there," a sprightly young nurse once exclaimed, who had just finished her training in an up-to-date training school. "We cannot accept your application unless you come as a probationer," a gentle, dignified matron would reply. Yet, in spite of all these seeming drawbacks, I consider my two years at the Longmore Hospital as not only the happiest, but also, so far as my technical knowledge of nursing is concerned, the most valuable in the whole of my nursing career. I arrived at the Hospital one bright May afternoon, and was shown at once into the matron's sitting-room. I was young and very timid, for I had struck out a new path in life, and taken my future into my own hands, amidst great opposition. I had no parents living whose authority I was violating, but other guardians thought the life most unsuitable; and, save for the help of a friendly doctor, I was entirely without sympathy. I looked at the matron; she was tall and dignified, with a quiet, gentle manner. She gave me some tea, and in five minutes I felt I had found a friend. I suppose she had faults like other people, but to me she was always perfect, and looking back on my two years' training under her, and on fifteen years' happy friendship (from the time I left the Hospital until her death), I still wonder what those faults were; and I cannot feel sufficiently grateful that I had the privilege of her careful training and the example of her beautiful unselfish life.

In those days the Hospital accommodated about fifty patients, and the preference was given to those who were in the latest stages of phthisis, heart disease, and cancer. We also had during the time I was there four patients suffering from a fractured spine and, as in all similar hospitals, several cases of paralysis and rheumatism. The staff consisted of the matron, five sisters, and two probationers. The sisters had all been trained in large general hospitals, and besides being the type of women suitable for this special nursing, were most efficient nurses. They were young, bright, and well educated, and each gave, as did the matron, the best years of her life for those who suffer most.

Four of the sisters had their permanent wards, and were on day duty; the fifth undertook the night work, and the probationers were on day and night duty for three months alternately. The day probationer was on duty alternate weeks with the two sisters who took charge of the cancer wards, as these were in addition to the large general ward and private ward of which each sister had charge.

Beyond dusting and keeping clean all utensils used by the patients, there were no menial duties, as the matron knew that the demands made by the patients on the time and patience of the nurse were very numerous; and we were taught from the first that to the nurse the personal welfare of the patients must be the first consideration.

Actual nursing was taught from the outset, and I have often met nurses who, after three years' training, had not had the opportunity of performing many duties which were quite usual to me by the time I had been in the Hospital three months. The giving of enemas, passing the catheter, washing out the stomach or bladder, and hypodermic injections were everyday occurrences, and the sister, only having one probationer, naturally allowed her to see and assist with all these duties from the first.

Another most useful items taught was not only the prevention of bed-sores, but also the curing of them, as very often patients were admitted who had been ill for a long time and who had not had the advantages of trained nursing. In every instance, notwithstanding the unfavourable condition of the patient, the sores would heal in a few weeks.

I consider, also, that it was a great privilege to have been a probationer in Edinburgh during the time that Professor Bell gave his clinical and surgical lectures to nurses. Those who have not had the advantage of those lectures, but who know that Professor Bell's personality gave Sir Conan Doyle his inspiration for his clever detective stories in which Sherlock Holmes takes so prominent a part, will understand the boon it was to the nurses to be carefully trained in the details of observation, which mean so much when nursing a critical case.

I have noticed lately that in an interview between the matron of one of the large London infirmaries and The Hospital Commissioner that the matron speaks with favour of those probationers who have received previous training in a children's hospital, and I cannot but think that when a "would-be probationer" is too young for training in a general hospital, a year or two spent in a hospital for incurables would be equally advantageous, especially if in after life she should become a private nurse. The absence of "rush" which necessarily goes on in a busy hospital prepares a nurse for the occasional monotony of private cases, and impresses upon her the importance of little attentions of which patients of the upper class think so much.

It is perhaps a little hard for a nurse to become a subordinate after having been trusted with seemingly more important duties, and yet the previous training will often have opened the eyes of the young nurse to see how necessary the so-called menial duties are for the efficient management of a perfectly-kept ward, and how much the nicety of their performance conduces to the comfort and well-being of the patients.

There is also to be considered the gain to the probationer's own character by witnessing the fortitude with which the patients bear their pain and affliction. It is wonderful to watch the daily development of all that is best in their nature, how cheerful and grateful they are, how they enter into the interests and pleasures of the nurses, and how they appreciate all that is done in the way of getting up concerts and entertainments for their amusement. For anyone who loves a life of service, rather than to one who aspires to a brilliant career, this special nursing will appeal. But even the more so-called "brilliant" nurse will never regret having given one or two of the best years of her life to help and cheer those who so depend upon others for any brightness that can be brought into their otherwise sad and often lonely lives.

### To Nurses.

**We invite contributions from any of our readers, and shall be glad to pay for "Notes on News from the Nursing World," or for articles describing nursing experiences at home or abroad dealing with any nursing question from an original point of view, according to length. The minimum payment is 5s. Contributions on topical subjects are specially welcome. Notices of appointments, letters, entertainments, presentations, and deaths are not paid for, but we are always glad to receive them. All rejected manuscripts are returned in due course, and all payments for manuscripts used are made as early as possible after the beginning of each quarter.**

### Making the Most of a Night off Duty.

BY A CORRESPONDENT.

I was to have a night off. A whole, long, dark, still night in bed; and only those whose labours compel them to turn night into day can realise what that means! The anticipation of a sleep unbroken by the ringing of bells, rumbling of traffic, yelling of newsboys, and the thousand and one noises which go to make the day of an ordinary individual, and the night of a night nurse. The anticipation of a night off under these circumstances is almost as good as the realisation—almost, not quite!

In order to enjoy the prospective night to the full, by getting completely tired out, I arranged with my chum to have a day's cycling in the Dart country, the beauty of which it is not possible to praise too highly. The banks of the Dart clad in the rich colouring of autumn, the magnificent slopes, the gleam of the water, and the repose which is over all, form a sight to be remembered, and in the rush and tear of a nurse's busy life, one to be treasured as an oasis in a desert of sickness and suffering.

We started off on a recent autumn morning. There was a tinge of frost in the air, which, combined with brilliant sunshine, was as good a tonic as one could wish for, and exhilarating to a degree. We pedalled along a coast road for some miles, passing through the pretty town of Paignton, with its wonderful stretch of sand, which in summer is dotted over with bathing tents, forming quite a mushroom-like colony nearly half a mile in length. From the high ground beyond Paignton we got a wonderful view across Torbay, with Torquay beautifully verdant and queenly, and quite Italian in aspect, rising on green slopes on the left and the heights of Berry Head, with the ancient town of Brixham, famous for its trawlers and its fishermen, on the right. We now began to wind inland, passing through several miles of beautifully-wooded country and through the picturesque village of Galmpton, finally arriving at Dittisham Ferry on the Dart. The river at this point is wide and winding. The surrounding woods looked magnificent in their autumn colourings, ranging from vivid green to warmest reds and browns, and we were greatly impressed with the beauty of the scene. Dittisham is a quaint village, with thatched cottages, and, viewed from the opposite river bank, is very pretty. Here, in order to cross, we had to ring a bell for the ferryman, who put off in a boat, and soon landed us, bicycles and all, on the Dittisham bank. The ferryman, at my request, proceeded to tell us how to reach Dartmouth, but the directions being given in the broadest Devon dialect, we were very little wiser when he finished. Dittisham is famous for its plum orchards. I asked one of the villagers if it were possible for us to buy some plums, and was informed with a pitying smile at my ignorance, that we certainly could not, as the trees were all stripped nearly a month ago. Abashed, we trudged with our bicycles up the stony steps of the village, regretting that life within hospital walls is not conducive to the remembering of times and seasons, especially the time and season of plum gathering.

The houses are quaint and smothered in foliage, and there is a wealth of flowers on all sides. An old lady gave us more explicit directions as to how to reach Dartmouth, and we started off, but found it almost impossible to ride through the Devon lanes. However, we trundled on, and, to make up for the want of plums, we found heaps of luscious blackberries, which we gathered and ate as we walked. Finally, after three hours pushing, trudging, and riding we arrived at Dartmouth. Here we lunched and had a good rest, which we both needed by this time, and here I could have enjoyed forty winks, but when I thought of the long-looked-for-night in bed which was coming, I banished all drowsiness, and we sallied forth to view the beauties of the neighbourhood. One of the bicycle tyres had sustained a puncture, which the cycle-mender informed us was caused by a "brimble." After much conjecture as to what a brimble might be, we learnt it was a bramble or blackberry thorn.

We duly admired the new naval college, which stands on a commanding eminence above the town, also the quaintness of the architecture of the houses, with the picturesque old castle and church nestling amongst the trees at the mouth of the river, and the village of Kingswear on the opposite bank.

Time was passing and we must get on. We chartered a boatman, and had ourselves and bicycles conveyed to the Kingswear side, passing the old cadet training ship *Britannia*, looking rather deserted now that the cadets have been transferred to the new college. There was a stiff breeze blowing as we crossed the river, bringing with it a little rain. We wondered if we were in for a shower, but the boatman remarked it was only a "scud" and would soon pass over. The "scud" combined with the "brimble" upset our gravity considerably.

From Kingswear we set off for Totnes, a distance of ten miles. The first two miles was a steady rise, but after gaining the high ground, we had a lovely run. In the distance could be seen the wide stretches of Dartmoor, with Haytor Rocks standing like sentinels on the horizon. Crossing the Dart again at Bridgetown, we entered the steep Totnes High Street, with its butter-walks, and overhanging houses, its beautiful old church, ruined castle, and ancient Guildhall. At Totnes we found friends and tea, and much needed clothes-brushes, for the Devon dust sticks closer than the proverbial brother. After a good rest, followed by dinner and a friendly game of whist, we again mounted our trusty cycles, and headed for hospital and bed, arriving happy, though thoroughly tired out.

Thanks to my long spell of wakefulness, combined with the pure Devon air, sleep required no wooing, and soon I was in the land of dreams. I enjoyed to the full my night off, and now I am looking forward with pleasurable anticipation to another day in the lovely Dart country, perhaps to be followed by another lovely night.

### Incidents in a Nurse's Life.

Contributions to this column are invited.

### THE MAGISTRATE'S ORDER.

During the last epidemic of small-pox in 1901 I was working as an ambulance nurse in the East End of London.

Many and varied were my experiences. The scenes of taking patients away from their friends were always painful. Sometimes they became violent and unpleasant, but more often they were sadly pathetic, for alas! they never knew if the separation might not be for ever.

One day I was waiting for instructions and a list of cases at a telephone station.

Suddenly a doctor opened the door of the ambulance and looked in.

"Nurse," he said, "I have an urgent case for you to remove at once. You will need to be very quick about it. The patient is a baby nine months old. You will find it on the mother's lap, surrounded by relatives. They are determined not to let the child go to the hospital, and will give us trouble. I have a magistrate's order in my pocket if they make a scene, and we shall be obliged to use it. But I think if you were to rush into the room, seize the child, and rush out again, it would be the best plan, and I will keep in the background with the order, and only produce it with compulsion." It seemed likely to be an exciting scene. I promised to do my best to get the child away, and begged the doctor to let me go into the house alone. He very kindly stood "on guard" outside, for the house was of evil repute.

Up the wooden broken stairs I passed into a room which was called the bedroom, the floor carpeted with fluff and dirt, the paper hanging in dank, torn pieces from the walls, the window of broken panes stuffed with rag and paper.

In the far corner sat a woman with wild, shining eyes and rough matted hair. She was surrounded by four women, all untidy, rough, and dirty. In the semi-darkness I could discern the figure of a child upon the mother's lap. As I went nearer I saw that the baby was very ill; so covered with the disease that the features were hardly distinguishable. The shining eyes of the mother gleamed fiercely at me as I knelt down and took the little one's hand within my own. I hardly knew what to do, what to say. Those eyes, with their fierce intense brilliancy seemed to awe me. They fascinated me. I saw the deep absolute love in them of a mother for her child, and the agonised look of an animal about to be robbed of her young.

"Poor little thing!" I cried, stroking the little hand which hung motionless. There was a slight stir and the women closed round me, as though they anticipated my escape with the little patient. I felt rather than saw the angry gleam from the mother as she drew the head of the little one nearer to her.

"Won't you let me take her where she will be well nursed and cared for? She is very very ill, poor little thing."

"No! she shan't go," cried the women in chorus; but the mother did not speak. She only gave one intense look of combined anger and sorrow.

I went on stroking the little hand, for I could not bring myself to attempt to take the almost dying child from those arms. There was a long silence, broken at last by a choking gasp from the little patient. For one moment the shining eyes looked away from me to the unconscious child.

Then she rose and suddenly held the baby towards me. Her eyes, though still bright and shining, were full of tears now.

"You shall take her," she cried in a shrill, heart-broken voice; "You shall take her if you'll promise on yer solemn oath that you'll nurse 'er yoursel!"

And with a sob that came from a heart full of despair she turned from me and flung herself on a mattress in the corner.

. . . . . . .

So the magistrate's order was not needed.

But twelve days after the mother herself was taken to the hospital suffering from the same complaint. Here to her wonder and her exceeding happiness she found her little one again almost well, smiling and contented. The gratitude of the woman was good to behold.

### Death in our Ranks.

A touching ceremony took place at the Portsmouth Infirmary on Friday last, when the remains of Nurse Louisa M. Westropp were laid to rest in the Kingston Cemetery. The deceased, who contracted typhoid fever, was a probationer in her third year and had won the love and respect of all with whom she had worked. The coffin, which was conveyed from the Nurses' Home by the male officers of the infirmary, was covered by the banner of St. John of Jerusalem and followed by the nurses, all of whom carried white flowers. Among the many beautiful wreaths sent were two from the patients in the wards where she had so lately worked.

### The Nurses' Bookshelf.

Lectures on Midwifery. By E. Stanley Hoare, M.R.C.S., D.P.H.Lond. (London: Scientific Press, 28 and 29 Southampton Street. Price 10s.)

These lectures are intended to meet the requirements of candidates for the Central Midwives Board examination. They would be more accurately described as syllabuses, and afford excellent outlines for the assistance of any qualified lecturer on midwifery. A considerable amount of amplification and simplification would be needed to make them clear enough for the average audience. Supplied with these syllabuses for reference the pupils would be freed from what is often the futile and misdirected effort of note-taking, and could give their undivided attention to lecturer and diagrams. The papers will also be of use to midwives who feel the need from time to time of refreshing their memory with regard to the more difficult and unusual parts of their work. The first three lectures are devoted to the elementary anatomy of the parts involved, followed by three on the signs of pregnancy, five on natural labour, and three on abnormal, and there are others on obstetric emergencies, puerperal fever, and the diseases which may affect infant life during the first ten days. Practical hints of considerable value are to be found here and there. For example, the reminder that the asistance of three persons is needed at a confinement. For want of this third person, Fetch and Carry, as the author names her, it not unfrequently happens that mother or child is temporarily neglected, and it may well be at some critical moment. Fuller's earth should not be recommended for infants as it is heavy and apt to cake. The most satisfactory dusting powder is made of two parts starch, one part zinc, and one part boracic, and even of this the less that is used the better. The twenty-four lectures are clearly printed on separate sheets and contained in a neat case 10¼ inches by 4. This is done that the pupil may study the points of one lecture at a time without the necessity of carrying a book about and searching for the information she requires. The idea is a new one. Diagrams which the pupils could study at leisure would be a valuable addition.

Dietetics for Nurses. By Julius Friedenwald, M.D., and John Ruhrah, M.D., of the College of Physicians and Surgeons, Baltimore. (Philadelphia and London: W. B. Saunders and Co. 12mo. 6s. 6d. net.)

This book is stated in the preface to be for "nurses and laymen interested in feeding the sick." There are few questions that a nurse could ask on the subject of feeding to which an answer could not be found here; but the book hardly seems suitable for amateurs, nurses or otherwise. The first few chapters are concerned with the chemistry and physiology of digestion, after which follows most valuable and well-arranged information on diet at different ages and in almost all classes of disease. The chapter headed "General Rules for Feeding the Sick" is one full of common sense, which cannot fail to commend itself to the experienced nurse, and to be of use to the beginner. It contains a good many hints for a fluid diet from which milk is to be excluded, a rather difficult diet to manage with full satisfaction to the patient. There are useful suggestions scattered about the book as to the use of milk, its various modifications and preparations; for instance, "If milk is given plain (to adults) it is only a question of time when it will disagree with any patient"; and again: "A most valuable method of preparing milk for invalids with whom it disagrees is to mix equal parts of milk and thoroughly cooked barley, rice, oatmeal, or arrowroot water, and boil them together for ten minutes." The authors have made ex haustive lists of diets for various intestinal disorders and for diabetics; and besides excellent instructions for feeding children, both in health and disease, there is a chapter on feeding the aged, from which we learn that after the age of fifty "the practice of eating heavy suppers late at night and of eating between meals should be discontinued"! Some persons would think these customs undesirable at any age. Fifty pages of suitable recipes contain many not to be met with in other cookery books for invalids, though no instructions are given for preparing zwieback, which is mentioned in many of the lists of special diets. This is certainly a book for every hospital to add to its nurses' library.

### St. Luke's Day at the Royal Hants County Hospital.

BY A WINCHESTER NURSE.

"St. Luke's Day" will always be remembered by those interested in the Royal Hants County Hospital at Winchester. Founded in 1736, it has long been the custom to have a special service in the beautiful chapel belonging to the hospital, and no pains have been spared to make the occasion a real pleasure to the 200 or more guests invited to attend. The entire building was last Wednesday thrown open from 3 P.M. till 6 o'clock, and on entering the front hall the first sight of the beautifully decorated staircase drew many an exclamation from the visitors; up the whole length the banisters were lightly and most artistically covered with autumnal tinted asparagus, interspersed with sprays of small mauve Michaelmas daisies, each staircase window being in keeping. The chapel at the top of the building was a mass of virginian creeper, palms, and chrysanthemums, with here and there small bunches of lily-of-the-valley perfuming the place with sweet scent. As the chapel filled many were obliged to remain outside the wide open doors, thus forming a double line down which filed the nursing staff singing a processional hymn. Quietly, and in perfect order they took their places in the chancel, and, looking at the pretty striped dresses of the nurses and dark blue of the sisters. The singing was most hearty, and the violin and 'cello accompanists were a great help. An appropriate address on the words "Only Luke is with me" was given by the Rev. W. G. Edwards, from Eastleigh, and the offertory was collected, the proceeds to go to the Samaritan Fund, which is benefited by nearly £15.

The service closed by a recessional hymn, and the guests dispersed to the various tea tables presided over by the sisters. After a sumptuous tea the wards were next visited and fully justified the care bestowed on them by the nursing staff. Everywhere there were flowers, plants, and bright faces and one almost forgot the pain that many were suffering on seeing how cheerful they looked. The great feature of the day was a visit to the Board-room, which was literally like a grocer's shop—the result of an appeal for "pounds." In every direction they were stacked with tea, sugar, rice, and, in fact, almost everything in the shape of food. Certainly, Winchester people have proved their generosity and the hospital is most grateful for all the gifts. Among the guests were many former nurses and sisters and two or three matrons who had not forgotten "St. Luke's Day." A general feeling of brightness and happiness prevailed and everyone was agreed that warm thanks were due to the popular matron, Miss Carpenter-Turner, and to her willing workers for their kindness in so ably carrying out the wishes of the committee in entertaining the general public.

### Everybody's Opinion.

[Correspondence on all subjects is invited, but we cannot in any way be responsible for the opinions expressed by our correspondents. No communication can be entertained if the name and address of the correspondent are not given as a guarantee of good faith, but not necessarily for publication. All correspondents should write on one side of the paper only.]

### A NURSE'S EXPERIENCE IN YORKSHIRE.

"Trained Nurse" writes: In the beginning of August I came into the neighbourhood of Harrogate and seeing one of the nursing homes advertising for nurses I applied. The matron wrote requesting me to call with testimonials, which I did; she asked me to leave them with her and she would take great care of them. I reluctantly did (they were originals). Three weeks passed, and hearing nothing from her, I called for my testimonials. After waiting forty minutes I was told matron was engaged and business must be attended to. I wrote asking her to send them, then a week later I called. She was out, so I left a message saying I would call next day if she would kindly leave them out for me. I did so and found her out again and no testimonials forthcoming. I called no more, but at the end of two months she sent them. During this time I had been in communication with a lady who wanted a trained nurse for night duty, while the untrained one took the day duty. She called to see me and said she would decide in a few days. A week later she wrote that she had engaged another untrained nurse and would be glad if I would go and put her into the way of things! Needless to say I declined. My last experience was on October 11. A friend and I went to Knaresborough, and we saw in the market place, standing on a barrow, a young woman, dressed in the uniform of the Great Ormond Street Hospital. She had a crowd of people round her, and was shouting her wares, "Tooth-powder and Sulphur Tablets," at the top of her voice, in opposition to a man at a crockery stall who called himself Bill Bailey. We spoke to her afterwards, and asked if she had ever been trained; she said, "Yes, in private, at Batley." We told her we were nurses. She kept saying, "It's all right, there's no harm done," even when we said we thought of writing to the hospital about her.

[How many more times will it be necessary to caution nurses under no circumstances to part with original testimonials?—Ed. The Hospital.]

### ONLY A FEVER NURSE.

"Untrained" writes: I should like to write a few lines to encourage "Contented" because I admire her spirit and good intentions. I often see advertisements in the papers to this effect, "Only gentlewomen need apply." I know that there are gentlewomen who take up nursing as a profession; but do all the women who answer these advertisements prove themselves to be gentlewomen either by their actions or by their conversation about their patients or fellow nurses? The word gentlewoman conveys different meanings to different individauls I suppose. The poorest, humblest woman God created can be a gentlewoman to her fellow creatures, and her gentleness should make her great, not her uniform nor her training in a "crack hospital." She need not have well-to-do friends or rings on her fingers, or high looks, but just a tender heart full of sympathy for the suffering creatures she is sent to minister to, and for the aged whose race is nearly run. Some nurses plead that their training hardens them; they certainly have to school themselves to witness very harrowing scenes, but that should not make them less gentle towards others. Trained nurses should be noble women, gentle and sympathetic in all their actions with all people. No doubt some people are very exacting and expect a great deal from them as if they were supernatural beings, but if each one would exhibit the spirit of gentleness there would not be so much friction. As an untrained nurse I have nursed all kinds of people suffering from all kinds of diseases (mental patients included). I have followed out doctor's directions as conscientiously as possible, and have never had any complaints from either doctors, patients, or maids during nine years of hard work. I must add that I wear a uniform for cleanliness and convenience, but not a veil. I daresay this letter will excite plenty of sarcastic remarks, but I am writing from experience, and because I think that all the petty strife and contention about such trifles a waste of time, and shows want of breeding, vulgarity, and bad feeling generally.

### NURSING IN SMALL ISOLATION HOSPITALS.

"Fever Hospital Matron" writes : I cannot help feeling that it is most unfair to cast the onus of the maladministration of some of our smaller isolation hospitals upon the governing body. If each joint hospital board were not a law unto itself it would often be a sorry day for the matron when that official is responsible for the distribution of beds —and the available ones few—with two or more districts indulging in an epidemic; each authority will consider their case the more urgent, and so it is—to them. As to medical officers being unable to get good nurses because the board will not pay good salaries, I have known a medical officer who, by his actions, evidently considered the servant who had to help with the nursing as well able to receive and carry out his instructions as the trained nurse-matron. The suggestion of exchanging nurses is not likely to work well, for nurses will never take kindly to such a scheme, and one could hardly expect them to. The employment of probationers has many points to recommend it; they do the minor menial work, which would be quite out of place for the nurse, and which, added to the rough work, would be too much for one wardmaid. Again, they are not free to leave the service of the hospital at their own sweet will, and by engaging one or two twice a year a preponderance of rawness at any one time is avoided. But surely few are paid so little as £10 or £12, for £15 or £20 is, I think, nearer the average. As joint hospitals are usually in out of the way places this fact may account for a difficulty in getting nurses to remain for long, but I fail to see that the same reason should account for matrons of inferior training and character being frequently employed. I believe that many small isolation hospitals are badly managed, but I doubt if the committee should always be blamed. I know an instance where a trained matron was appointed to succeed caretakers. Everything was in a deplorable state. The doctor who visited condoled with the matron, and, with others, assured her that the board was so parsimonious that poor Mrs. —— (the late caretaker) could not get things necessary to work the place ; on the other hand, one or two members of the board called assuring the matron of their desire for efficient work, and promising their support. They soon proved that the caretakers had done a gross injustice to the board, whose desire it had ever been to secure the best nursing and treatment for the patients in their hospital. Strict economy must be studied in a rate-supported hospital, but I question any board being so bad as they are represented if they were handled in a judicious manner, and the facts and possibilities of the case pointed out to them. If a joint board rejects a proposal to instal hot-water pipes to supplement open fires, they but carry out the recommendations of the Local Government Board, whose various rules and restrictions are irritating to many, but are all framed upon a sound and practical basis. With regard to the desirability of separating acute from convalescent cases of scarlet fever, it is not always possible; it sometimes happens that, owing to an epidemic, the acute beds are all filled, and new cases must be put in the convalescent wards. This may happen in a hospital with fifty or sixty scarlet-fever beds. In conclusion, I should like to point out that the work of a matron in a joint isolation hospital must always be of a most arduous nature. She cannot have day and night staff waiting in readiness for diphtheria cases, which are rare in the district, or for small-pox, which, perhaps, comes about twice a year. But she must have the wards in absolute readiness, and she must also be able to draw the staff, when required, from some other wards. She must never leave the hospital without giving instructions as to who is to take certain duties should fresh diseases need admission during her absence, and nothing must interfere with the prompt removal of each case as it arises. Perhaps I have had an unusual experience, for although I have gone into some deplorable hospitals, I have always gained the support and confidence of any board I have served under, and been allowed to organise in a proper manner.

### NURSING ASSOCIATIONS IN NATAL.

"Another Who has Tried" writes : Permit me to endorse all that "One Who Tried" writes in your issue of August 12. I for one joined a Co-operation, the charges being as follows : Bed, 1s. 6d. per night, or 10s. per week; meals, 1s. 6d. each, or 4s. per diem; wardrobe, £1 per annum; stockbox, 15s. per annum; club subscriptions, 5s.; nurses' subscriptions, 10s. 6d., plus 5 per cent. on all fees from cases, whether got per Co-operation or from your own connection. I was urged to give up my home here by the superintendent and live in the Nurses' Home, as I reside twenty miles from Durban. One day in the Nurses' Home was enough to prove to me that peace or opportunity for rest was quite out of the question. Meals were hurried in and hurried over, the superintendent hastening off out of the house before anyone had finished. A very gorgeous sitting-room had two screens at the end of the room, behind each of which was a bed and bedroom accessories. I never got a case from or through the Association. Fees were, and are, 4 guineas per week, which pans out at 6d. per hour, for surgical, medical, and maternity cases; and 5 guineas per week for mental; all of which I have nursed. During the war trained nurses from home were not to be got, and I therefore was readily snapped up and kept busy. Now there are myriads, who far outnumber the available cases. I have been disengaged for eight weeks and have no case in view. The fact is that nurses are advertising to take work at half fees. Then there are a considerable number of Sarah Gamps, who take small fees and possibly give satisfaction to their clients who are accustomed to them and regard the modern trained nurse as a faddist. For instance, in serious cases of enteric, maternity, etc., the doctor forbids company. Nurse, if trained, endeavours to carry out his instructions. But bedrooms are invariably on the ground floor and within two or three feet of the front-door, which always stands open by day. Nurse has, in addition to sick-room duties, to perform sundry others outside, such as preparing her patient's food in a kitchen built usually quite separate from the house, and on her return, hoping to find the patient quietly asleep, it is a common experience to discover a visitor talking loudly and continuously. If there is a baby it has been taken out of bed and is being violently jogged, and this probably within three or four hours of the event. If the nurse reminds visitors that the doctor has forbidden callers and asks them to go into another room, she gives dire offence. "They have always done it before; why not now? Such nonsense!" etc. I give one case in point—a maternity case, where there had been grave fears for the life of the mother from antepartum hæmorrhage and of child from uterine inertia. The patient had fourteen or fifteen relatives living near, and all and every one, including girls of thirteen years, expected to be admitted and to talk unrestrainedly in the sick room. Strange to say, though one member had nearly lost her life from the same cause, she was the worst offender. Of course the pulse and the temperature told their own tale, and the nurse had to pay the penalty in extra fatigue and anxiety as well as the patient. All servants are Kaffir boys or men, or if on sugar or tea estates, coolies, who are frequently what is called here "raw," that is, they have never before worn civilised clothes nor worked in a white person's house. Usually a Kaffir foresees an illness and clears out just at the critical time, so that a raw one has to be got to make shift. Such a one is a very unclean creature, and drops all over the place unmentionable creatures, which nurse finds creeping over her white apron or ensconced in all the folds of her clothing. Then, too, he has an aptitude for always leaving the gruel or milk saucepan dirty, and for letting the fire out, so that no hot water can be got. The stoves are American and burn wood, and if hot food or water is wanted in the night nurse has to go out of doors to the kitchen and probably hunt for kindling. If she has a dislike to cockroaches—which in this country overrun everything, are in every room, on beds and walls, and smell vilely—these nocturnal duties are not pleasant ones. There is another evil here : except in big towns like Durban and parts of Maritzburg, the sanitary arrangements are very primitive. A roughly-built outhouse, with an ordinary zinc pail, serves the purpose. There are seldom any disinfectants or deodorizers, and the outhouse is usually situated 100 to 120 yards from the house. Day and night, in all weathers, nurse has to walk there through long grass or ankle-deep in sandy dust or mud, perhaps under trees from which occasionally a snake hangs from a bough, and surrounded by myriads of great toads and enormous hairy spiders, such as could not be even imagined at home. Further, in maternity cases it is usual to engage the nurse for only a fortnight, and probably she has to go from one to three days' journey to get to her next case. Almost invariably the nurse has to sleep in the same room—that a small one—with her patient, who often objects to open window or door at night. The short time of nursing, in my opinion (as well as in that of many doctors here) is responsible for the vast number of women one meets who have undergone, or must undergo, ovarian operations. At one severe case of enteric—in a very small room—my bed was within 3 feet of the patient's, and though the parents are nice people they allowed me to be without rest for seven nights and days until the two doctors insisted on someone being got to relieve me while I got a night's rest and sleep. The little patient was very wilful and the parents could not manage her at all, and though perforation of the bowel was feared, the mother was so desirous of raising the child into a sitting posture that I dared not leave her. In addition a smaller child was running in and out perpetually with bread and toast in its hand; which the patient would have eaten if I had not been constantly there. So it will be seen that nursing is not child's play in this trying, hot, humid climate. I had been nursing at home for a good many years, but I have come to the conclusion that nursing is infinitely easier and pleasanter work at home than here, either in Natal, Pretoria, or other parts of the Transvaal or Zululand. With regard to your remark that poverty is only comparative here, bricklayers, masons, etc., get from 2s. 6d. per hour, but everything is at least half or two-thirds dearer here than at home. If what I have written will deter any nurses from coming out here to starve or worse I shall feel justified, and trust you will find room for this in your valued and eagerly looked-forward-to paper.

### Appointments.

[No charge is made for announcements under this head, and we are always glad to receive and publish appointments. The information, to insure accuracy, should be sent from the nurses themselves, and we cannot undertake to correct official announcements which may happen to be inaccurate. It is essential that in all cases the school of training should be given.]

Harton Hospital, South Shields, Newcastle.—Miss E. J. Helliwell has been appointed sister. She has been assistant nurse at West Grove Home, Halifax, Yorkshire, and was trained at Leeds Infirmary. She has also done private nursing in Southsea.

Livingstone Hospital, Dartford.—Miss Dora Weaver has been appointed staff nurse. She was trained at the Wolverhampton General Hospital, and has since been doing private nursing. She holds the certificate of the Central Midwives Board.

Milnthorpe Workhouse Infirmary.—Miss K. Mohan has been appointed head nurse. She was trained at the Sculcoates Union Infirmary, Hull, where she remained for six months after completion of training. She has since been charge nurse at the Deanhouse Union Infirmary, Holmfirth, and nurse matron at the Hollin Heys Small-pox Hospital, under the Elland District Council. She has also done private nursing.

National Hospital for the Paralysed and Epileptic, Bloomsbury.—Miss Isabel Lawrence has been appointed lady superintendent. She was trained at the Crumpsall Infirmary, Manchester. She had charge of male and female wards at the Cancer Hospital, Brompton, and was subsequently charge nurse and sister at the National Hospital for the Paralysed and Epileptic. She has also done private nursing, and has since been lady superintendent of the Meath Home of Comfort for Epileptics at Godalming.

Poor Law Infirmary, Eastville, Bristol.—Miss Mary A. Cayes and Miss Ethel Rose have been appointed charge nurses. Miss Cayes was trained at Birmingham Union Infirmary. Miss Rose was trained at Bradford Union Infirmary, and has since been attached to a nursing home at Harrogate.

Southwark Infirmary, East Dulwich Grove.—Miss E. Bolland has been appointed sister. She was trained at Clayton General Hospital, Wakefield, and has since been sister at Stockton and Thornaby Hospital.

Stafford Isolation Hospital.—Miss Sara Bridge has been appointed nurse matron. She was trained at Staffordshire General Infirmary, where she has since been night sister. She has also been night superintendent at Blackburn and East Lancashire Infirmary.

### Presentations.

Hartford Parish.—An interesting presentation took place in the parish of Hartford, when Nurse Brookes, who is retiring from district work to take up private nursing, was presented with a writing desk contributed to by many poor patients and friends. The "mothers" gave a tea in the mission room, to which they invited Nurse Brookes, who also received many private presents from patients and friends. She has worked in Hartford for nearly six years.

Limerick County Infirmary.—Miss Janet Mayne, matron of Limerick County Infirmary, has been presented with a heavy purse of sovereigns by the Chairman of the County Council, as a slight token of the appreciation by all classes of the people of Limerick city and county of the ability which she has shown, and of thankfulness to her for her untiring zeal for the good of the patients entrusted to her charge during the last eleven years.

### Where to Go.

Lectures to Midwives under the London County Council.—October 27, "Foxhill," Plumstead Common Road, S.E., 5.30 to 7.30 p.m., Dr. Lampert; "Millbank," Erasmus Street, Westminster, S.W., 7 to 9 p.m., Dr. Rocke. Monthly nurses are admitted to these classes and any further information may be obtained from the Secretary, Association for Promoting the Training and Supply of Midwives, Dacre House, Dean Farrar Street, Westminster, S.W.

### Wants and Workers.

Will anyone having a pattern of a nurse's cloak, with sleeves and detachable cape, kindly lend it to District Nurse, Cawston Lodge, Haverland, Norwich?

Through misfortune a married nurse has to return to private nursing, and would be very grateful for a cloak or other uniform of any nurse who has no further use for it. Address: Nurse Annie, 26 Southwater Road, St. Leonards-on-Sea.

### TRAVEL NOTES AND QUERIES.

By our Travel Correspondent.

Egypt to Rome via Naples (F. F. H.).—From Cairo to Rome by the Italian line via Alexandria and Naples, first class, £11 19s. 10d.; second class, £8 7s. 9d. From Cairo to Rome by Messageries Maritimes, via Port Said and Naples, first class, £14 0s. 7d.; second class, £9 15s. 3d. This sum includes landing and embarking fees. If you want more help let me know.

### Notes and Queries.

**REGULATIONS.**

**The Editor is always willing to answer in this column, without any fee, all reasonable questions, as soon as possible.**

**But the following rules must be carefully observed.**

**1. Every communication must be accompanied by the name and address of the writer.**

**2. The question must always bear upon nursing, directly or indirectly.**

**If an answer is required by letter a fee of half-a-crown must be enclosed with the note containing the inquiry.**

*Justice.*

(21) Is a matron justified in refusing to pass a probationer in her examination through one night duty report when she has had previous good reports from other wards?—*S. M. C.*

We cannot answer this question without knowing full details.

*Dispensing.*

(24) Will you please tell me whether it would be possible to learn dispensing whilst working in a hospital, and the best and cheapest way of doing so?—*R. M.*

Unless you were in a hospital where a course of instruction in dispensing is part of the curriculum, you will find it very difficult to learn dispensing thoroughly in your spare time. Miss Buchanan, Gordon Hall, Gordon Square, W.C., has arranged some special elementary dispensing classes for nurses.

*Fever Training.*

(25) I should be very glad if you would tell me whether it is advisable to enter one of the fever hospitals under the Metropolitan Asylums Board? I am thirty years of age, and cannot see my way to enter as probationer for the three years' general training. Should I be considered trained after a certain time in fever nursing?—*A. M. B.*

Training in a fever hospital alone would merely qualify you for fever nursing.

*Nursing in the Navy.*

(26) Will you please tell me where I can obtain particulars about nursing in the Navy?—*M. A.*

From the Director-General, Medical Department of the Navy, The Admiralty, 18 Victoria Street, London, S.W.

*Massage.*

(27) I am anxious to have the necessary training to obtain a certificate for massage, with the object of earning my livelihood as a professional masseuse. I have my first aid certificate of the St. John's Ambulance Association, and a slight knowledge of nursing. Could you recommend me a good school or teacher, and tell me the best way to obtain employment after training?—*R. M. H.*

Write to the Incorporated Society of Trained Masseuses, 12 Buckingham Street, Strand, London, W.C.

*Cancer.*

(28) Can you give information as to a home where a patient (gentleman) suffering from cancer in the throat can be received? A small amount per week can be paid. In or near London preferred.—*A. B. C.*

Write to the Friedenheim Hospital, Upper Avenue Road, Swiss Cottage, London, N.W., or St. Luke's House, 14 Pembridge Square, Bayswater.

*Indian Army Nursing Service.*

(29) Where should I apply for an appointment in the Indian Army Nursing Service? I am twenty-one years of age. Am I too young? What qualifications are required?—*B. S. R.*

Candidates must be fully trained nurses between the ages of 25 and 35, so you see that your age is an insuperable barrier.

*Instruction in Nursing Children.*

(30) Where could a girl obtain experience in nursing babies? The inquirer has had two years' experience in nursing in a sanatorium, but now has the offer of taking a baby from the month, and wishes to obtain three months' experience in any institution, either free or as paying pupil, before undertaking the responsibility.—*Miss B. A.*

Write to the Hon. Matron, Our Lady's House for Homeless Babes, 93 Shaw Street, Liverpool, or to the Matron, the Norland Institute, 7 Pembridge Square, Bayswater, W.

**Handbooks for Nurses.**

| | Post Free. |
|---|---|
| "How to Become a Nurse: How and Where to Train." | 2s. 4d. |
| "Nursing: its Theory and Practice." (Lewis.) ... ... | 3s. 6d. |
| "Nurses' Pronouncing Dictionary of Medical Terms." ... | 2s. 0d. |
| "Complete Handbook of Midwifery." (Watson.) ... | 6s. 4d. |
| "Preparation for Operation in Private Houses." ... | 0s. 6d. |

Of all booksellers or of the Scientific Press, Limited, 28 & 29 Southampton Street, Strand, London, W.C.

### For Reading to the Sick.

THE COMMUNION OF SAINTS.

My soul did build
To music of its own, a temple filled
With worshippers beloved that hither drew
In silence; then I thirsted not to hear
The voice of any friend, nor wished for dear
Companion's hand firm clasped in mine; I knew
Had such been with me, they had been less near.

*Dora Greenwell.*

Why should not those who are gone be actually nearer us, nor farther from us, in the heavenly world, praying for us, and it may be influencing and guiding us in a hundred ways of which we, in our prison-house of mortality, cannot dream? Yes! Do not be afraid to believe that he whom you have lost is near you, and you near him, and both of you near God, Who died on the cross for you.

And so with those who are Christ's whom we love. Partakers of His death, they are partakers of His resurrection. Let us believe the blessed news in all its fulness, and be at peace. A little while and we see them, and again a little while and we do not see them. But why? Because they are gone to the Father, to the Source and Fount of all life and power, all light and love, that they may gain life from His life, power from His power, light from His light, love from His love; and surely not for nought. Surely not for nought. For if they were like Christ on earth, and did not use their powers for themselves alone; if they are to be like Christ when they see Him as He is, then, more surely, will they not use their powers for themselves, but as Christ uses His, for those they love.

"Thy will be done on earth as it is in heaven." In heaven, my friends, as well as on earth. Form your own notions, as you will, about angels, and saints in heaven, for everyone must have some notions about them, and try to picture to himself what the souls of those whom he has loved and lost are doing in the other world; but bear this in mind, that if the saints in heaven live the everlasting life, they must be living a life of usefulness, of love, and of good works. Ask —"Father, Thy will be done on earth as it is in heaven." For in asking that, we ask for the best of all things. We ask for the happiness, the power, the glory of saints and angels. We ask to be put in tune with God's whole universe, from the meanest flower beneath our feet to the most glorious spirit whom God ever created.—*Charles Kingsley.*

Temporal death, therefore, can have no power to dissolve this holy fellowship. The members of Christ, who have been removed from our sight by death, still remain true and living members of Christ's mystical Body, only they have reached the point which conceals them from our view. Their trials are over, and they are safe in the world of spirits, in the presence of their Divine Head. There they hold communion with us, and we on earth with them. Like a great cloud of witnesses they encompass us about to encourage us in our heavenward path.—*Dean Hook.*

God calls our loved ones, but we lose not wholly
What He hath given;
They live on earth, in thought and deed, as truly
As in His heaven.

*Whittier.*

